# Isolation of *Francisella tularensis* and *Yersinia pestis* from Blood Cultures by Plasma Purification and Immunomagnetic Separation Accelerates Antibiotic Susceptibility Determination

**DOI:** 10.3389/fmicb.2017.00312

**Published:** 2017-02-28

**Authors:** Ronit Aloni-Grinstein, Ofir Schuster, Shmuel Yitzhaki, Moshe Aftalion, Sharon Maoz, Ida Steinberger-Levy, Raphael Ber

**Affiliations:** ^1^Department of Biochemistry and Molecular Genetics, Israel Institute for Biological ResearchNess-Ziona, Israel; ^2^Department of Infectious diseases, Israel Institute for Biological ResearchNess-Ziona, Israel

**Keywords:** *Francisella tularensis*, *Yersinia pestis*, bacteremia, immunomagnetic separation, antibiotic susceptibility, blood culture

## Abstract

The early symptoms of tularemia and plague, which are caused by *Francisella tularensis* and *Yersinia pestis* infection, respectively, are common to other illnesses, resulting in a low index of suspicion among clinicians. Moreover, because these diseases can be treated only with antibiotics, rapid isolation of the bacteria and antibiotic susceptibility testing (AST) are preferable. Blood cultures of patients may serve as a source for bacteria isolation. However, due to the slow growth rates of *F. tularensis* and *Y. pestis* on solid media, isolation by plating blood culture samples on proper agar plates may require several days. Thus, improving the isolation procedure prior to antibiotic susceptibility determination is a major clinically relevant need. In this study, we developed a rapid, selective procedure for the isolation of *F. tularensis* and *Y. pestis* from blood cultures. We examined drop-plating and plasma purification followed by immunomagnetic separation (IMS) as alternative isolation methods. We determined that replacing the classical isolation method with drop-plating is advantageous with respect to time at the expense of specificity. Hence, we also examined isolation by IMS. Sub-localization of *F. tularensis* within blood cultures of infected mice has revealed that the majority of the bacteria are located within the extracellular fraction, in the plasma. *Y. pestis* also resides within the plasma. Therefore, the plasma fraction was isolated from blood cultures and subjected to an IMS procedure using polyclonal anti-*F. tularensis* live vaccine strain (LVS) or anti-*Y. pestis* antibodies conjugated to 50-nm nano-beads. The time required to reach an inoculum of sufficient bacteria for AST was shortest when using the plasma and IMSs for both bacteria, saving up to 2 days of incubation for *F. tularensis* and 1 day for *Y. pestis*. Our isolation procedure provides a proof of concept for the clinical relevance of rapid isolation for AST from *F. tularensis-* and *Y. pestis*-infected patients.

## Introduction

*Francisella tularensis* is a gram-negative facultative intracellular bacterium and the causative agent of tularemia (McCoy and Chapin, 1912). As one of the most infectious pathogenic bacteria known, it is classified by the CDC as a Tier 1 select agent^1^. The disease is endemic in North America and Europe (Sjöstedt, 2007). Disease severity is influenced by the route of infection and the bacterial subtype. The most severe form of tularemia is caused by *F. tularensis* subsp. *tularensis* (type A), which is found in North America (Olsufjev and Meshcheryakova, 1983; Staples et al., 2006) whereas *F. tularensis* subsp. *holarctica* (type B) is responsible for tularemia across the entire Northern Hemisphere (Jusatz, 1961). *F. tularensis* live vaccine strain (LVS) (Eigelsbach and Downs, 1961; Tigertt, 1962) is an attenuated type B strain that does not cause disease in humans. LVS is virulent in mice and is thus used in mouse models of the disease (Sjöstedt, 2007). This vaccine has failed to achieve licensing by regulatory authorities (Oyston, 2009). Six clinical forms are observed: ulceroglandular, glandular, oculoglandular, oropharyngeal, typhoid and pneumonic (Anda et al., 2007). The latter is the most relevant in the context of bioterrorism because symptoms develop 3–5 days post-exposure and mortality rates reach 60% (Gill and Cunha, 1997).

*Yersinia pestis* is the causative agent of plague, a severe and rapidly progressing disease. Due to its extreme lethality and potential for aerosol transmission, *Y. pestis* is designated by the CDC as a Tier 1 select agent^1^. Symptoms usually begin 1–8 days post-exposure, depending on the route of exposure, infecting strain, disease form (mainly bubonic, septicemic, or pneumonic), dose of infection, and patient immunocompetence (Inglesby et al., 2000). Bacteremia is frequently observed in patients and infected animals (Butler et al., 1976; Perry and Fetherston, 1997). High mortality rates are observed if treatment is not started within 18–24 h after symptom onset (Pollitzer, 1954; Inglesby et al., 2000).

Both tularemia and plague are treated with antibiotics; no safe and efficient vaccines are currently available for tularemia (Boisset et al., 2014) or plague (Pechous et al., 2016). Unfortunately, highly fluoroquinolone-resistant mutants of *F. tularensis* are easily and quickly obtained (Sutera et al., 2014b), and some exhibit cross-resistance to other clinically relevant antibiotic classes. Similarly, although most naturally occurring *Y. pestis* strains are susceptible to recommended antibiotics, plasmid-mediated single and multiple drug-resistant strains have been isolated from patients (Galimand et al., 2006). Due to the severity of infection by *F. tularensis* or *Y. pestis* and the possibility of acquired resistance to recommended antibiotics (either naturally or intentionally), treatment should not rely solely on organism identification but should include antimicrobial susceptibility testing (AST) of the bacteria isolated from the patient. The conditions for performing AST for *F. tularensis* or *Y. pestis* are defined by the CLSI guidelines (CLSI, 2015) and are based on the microdilution technique. Defined concentrations of a bacterial suspension are inoculated into a series of twofold dilutions of the tested antibiotic in a relevant medium and incubated. The Minimal Inhibitory Concentration (MIC) is defined as the lowest antibiotic concentration that completely inhibits bacterial growth, as determined by the unaided eye, following incubation for 24 h (*Y. pestis*) or 48 h (*F. tularensis*).

Blood cultures are a major source of bacterial isolates for AST. In the past, the isolation of *F. tularensis* from the blood of infected patients has rarely been documented, possibly due to the poor sensitivity of blood culturing systems or the occurrence of bacteremia in the acute stages of infection (Pittman et al., 1977). However, the number of reported tularemia bacteremia cases has increased with the use of improved blood culture isolation systems (Reary and Klotz, 1988), and in recent years, more cases of tularemia bacteremia have been reported in the literature (Haristoy et al., 2003; Sarria et al., 2003; Khoury et al., 2005; Fritzsch and Splettstoesser, 2010; Mohamed et al., 2012; Karagoz et al., 2013; Larssen et al., 2014; Nirkhiwale et al., 2015; Ughetto et al., 2015; Briere et al., 2016). Furthermore, studies have reported bacteremia in mice infected intranasally with *F. tularensis* LVS 48 h post-infection (Chiavolini et al., 2008). The bacteremic phase lasts up to day 7 post-infection. In experimental respiratory tularemia in African green monkeys and cynomolgus macaques, bacteremia was detected 4 days post-infection (Glynn et al., 2015). The clinical significance of *F. tularensis* bacteremia remains unknown; however, bacteremia has been associated with a severe form of the disease and with compromising pneumonia (Karagoz et al., 2013). Bacteremia has also frequently been observed in plague patients (Butler et al., 1976; Perry and Fetherston, 1997).

Although bacteremia has been identified in both diseases, direct transfer of the blood culture into microdilution AST is not feasible, mainly because blood components may interact with either the bacteria or the antibiotics and influence the test results. Moreover, blood components do not allow optical quantification of the bacterial culture. Thus, bacteria isolation by sub-culturing on agar media is a compulsory step that is time-consuming due to the slow growth rates of these bacterial species. For *Y. pestis*, 1–3 days are usually required for the blood culture to be identified as positive, 2 days are required for colony isolation, and another 24 h are required for AST (Perry and Fetherston, 1997; Inglesby et al., 2000). Due to the slower growth rates of *F. tularensis*, up to 7 days are required for positive blood culture identification (Provenza et al., 1986; Haristoy et al., 2003), 2–3 days of incubation are required for the colony isolation step, and 48 h are required for AST to determine proper MIC values. In total, 6 days are usually required for *Y. pestis*, and 7 days or more are required until *F. tularensis* blood cultures are identified as positive and bacteria are quantified for AST (Fritzsch and Splettstoesser, 2010; Karagoz et al., 2013; Ughetto et al., 2015). In light of these time intervals, the development of rapid isolation procedures is needed to ensure the clinical relevance of AST results.

Previously, we reported that rapid separation of *Y. pestis* from blood components can be achieved using a serum separation tube (SST) (Steinberger-Levy et al., 2007). In another study, we have shown that an immunomagnetic separation (IMS) procedure can be used to pre-enrich *Y. pestis* from environmental samples (Zahavy et al., 2012). To reduce the time interval needed to reach quantified *F. tularensis* and *Y. pestis* cultures suitable for AST, we examined whether the application of an SST and IMS facilitates isolation compared to classic plating or drop-plating directly from blood cultures and whether these procedures can be implemented for the isolation of *F. tularensis* and *Y. pestis* from blood cultures.

We first characterized the bacteremic phase in *F. tularensis*-infected mice and validated *ex vivo F. tularensis* spiking of blood cultures as a mimic of infected blood cultures. Then, we compared the efficacy of the three isolation procedures for both bacterial species. The SST-IMS procedure enabled the isolation of sufficient amounts of *F. tularensis* for AST within the shortest time period for all blood cultures examined, ranging from approximately 10^3^ CFU/ml to greater than 10^9^ CFU/ml. Moreover, the SST-IMS method was also advantageous for *Y. pestis*, which exhibits faster growth rates than *F. tularensis*, suggesting that this method may be applicable to a wide range of bacteria.

## Materials and Methods

### Bacterial Strains, Media, Growth Conditions, and Colony-Forming Unit Determination

*Francisella tularensis* LVS (ATCC 29684) was grown on CHA agar (5.1% cystine heart agar, 1% hemoglobin), (BD Difco, Sparks, MD, USA) or in cation-adjusted Mueller-Hinton broth (CAMHB) (BBL Difco, Sparks, MD, USA, #212322) supplemented with 2% defined growth supplement (IsoVitaleX Enrichment; BBL Difco, Sparks, MD, USA, #211876) and 3 μM hematin (Sigma, Israel #3281) (termed HLMHI) at 37°C. The *Y. pestis* vaccine strain EV76 (Girard’s strain) (Ben-Gurion and Shafferman, 1981) was grown on BHI-A (brain heart infusion agar, BD Difco, Sparks, MD, USA, #241830) plates at 28°C. Colony-forming unit (CFU) counts were determined by plating 100 μl of serial 10-fold dilutions in sterile phosphate-buffered saline (PBS, Biological Industries, Beth Haemek, Israel) on CHA and BHI-A plates for *F. tularensis* and *Y. pestis*, respectively. Drop-plating was performed by plating 10 μl of serial 10-fold dilutions in triplicate on CHA and BHI-A plates.

### Animal Infection and Blood Collection

The animal experiment reported here was approved by the Israel Institute for Biological Research animal care and use committee and was conducted in accordance with the Animal Welfare Act. *F. tularensis* LVS bacterial cultures were grown to mid-log phase (optical density of 0.1–0.2 at 660 nm) at 37°C in TSBC (TSB Difco, Sparks, MD, USA, #211825 supplemented with 0.1% cysteine). The bacteria were washed and re-suspended in PBS at the desired concentration. BALB/c female mice (8–10 weeks old, Charles River) were anesthetized with a mixture of 60 mg/kg ketamine HCl and 10 mg/kg xylazine and infected intranasally by application of 25 μl containing 10^5^ CFU of *F. tularensis* LVS (10LD_50_). Mice were monitored daily for clinical symptoms.

At the blood sampling time points, mice were anesthetized with 150 mg/kg ketamine and 15 mg/kg xylazine and terminally bled by cardiac puncture using a heparinized syringe. Blood samples were pooled to a final volume of 2–5 ml.

### Blood Cultures

Two to five milliliter of fresh infected blood or 10 ml of human blood spiked with bacteria at defined concentrations was incubated in BACTEC Plus Aerobic/F Culture vial (BD, Sparks, MD, USA, #456005). The blood cultures were shaken at 150 rpm at 37°C in a New Brunswick Scientific C76 water bath for the indicated time periods.

### Fractionation of Blood Culture Samples

Blood culture samples were fractionated using SSTs (Vacuette Z serum Sep. Clot activator, Greiner bio-one, Kremsmunster, Austria #455071) to obtain the plasma fraction. The sample was loaded into the SSTs and centrifuged for 10 min at 1700 *g* at 20°C. For *F. tularensis*, the upper fraction was collected, and the bacteria lying on the gel matrix were recovered and added to the plasma fraction. For *Y. pestis*, the upper fraction was discarded, and the bacteria lying on the gel matrix were recovered with 1 ml of PBS. Mononuclear cells were purified from blood culture samples using Vacutainer CPTs (cell preparation tubes) NC (BD, Sparks, MD, USA, #362781) and Histopaque^®^-1077 gradient (Sigma, Israel #10771) following the manufacturer’s protocols.

### Immunomagnetic Separation

#### Preparation of Antibody Nano-Bead Conjugates

A 50-μg quantity of polyclonal rabbit anti-*F. tularensis* LVS serum (Mechaly et al., 2016) or 100 μg of polyclonal rabbit anti-*Y. pestis* Kimberley53 (Zahavy et al., 2012) were covalently linked to 10 mg of 50-nm magnetic beads (fluidMAG-CMX 4106-1, Chemicell, Gmbh, Berlin, Germany) as recommended by the supplier (protocol A11) using EDC (1-ethyl-3-[3-dimethylaminopropyl]carbodiimide hydrochloride) (Sigma, Israel #PG82079) dissolved in MES buffer (0.1 M 2-(*N*-morpholino)ethanesulfonic acid), pH 5.

#### IMS Procedures

A capture solution containing 80 μl of the polyclonal antibody-nanoparticle conjugate, 1% BSA, and 0.5% Tween 20 was added to the plasma fraction containing the isolated bacteria from the spiked blood cultures. The samples were incubated for 20 min with gentle rotation at room temperature. LS separation columns (Miltenyi Biotec. Inc, Auburn, CA, USA, #130-042-401) were placed on the MidiMACS magnetic separator (Miltenyi Biotec Inc, Auburn, CA, USA, #130-042-302) and preconditioned with 5 ml of PBS. Then, the sample was passed through the column. The column was washed with 3 ml PBS and removed from the magnet, and retained bacteria were washed off with 2 ml of HLMHI for *F. tularensis* LVS or 1 ml of BHI for *Y. pestis* EV76 pressed through the column with a piston. The IMS output was divided into aliquots of 100 μl in a 96-well microtiter plate. Bacteria were grown to O.D._630 nm_ = 0.1 for *F. tularensis*, which is equivalent to approximately 1–2 × 10^9^ CFU/ml, and O.D._630 nm_ = 0.035 for *Y. pestis*, which is equivalent to 2–3 × 10^8^ CFU/ml, using O.D._630 nm_ at time zero as a blank.

### MIC Determination

Microdilution antibiotic susceptibility standard tests were performed according to the CLSI guidelines for *F. tularensis* and *Y. pestis* (CLSI, 2015). The recovered bacteria were diluted to yield approximately 1–2 × 10^6^ CFU/ml in HLMHI for *F. tularensis* or approximately 10^6^ CFU/ml in CAMHB for *Y. pestis*, and 50 μl was inoculated in duplicate into 96-well microtiter plates containing 50 μl of twofold serial dilutions of each antimicrobial agent in the appropriate medium: doxycycline (Sigma, Israel #D9891), ciprofloxacin infusion bag (Teva, Petah-Tikva, Israel). The inoculum size was verified by plating 10-fold dilutions on CHA or BHIA and incubation for 3 days at 37°C or 2 days at 28°C for CFU counts of *F. tularensis* or *Y. pestis*, respectively. The microtiter plates were incubated in a plate reader (TECAN Infinite 200) for 48 h at 37°C for *F. tularensis* or 24 h at 28°C for *Y. pestis* in ambient air, and O.D._630 nm_ was read at 1-h intervals.

Etest assays for *Y. pestis* were conducted on MHA [Mueller Hinton Agar (BBL Difco, Sparks, MD, USA, #225250)] and on HIMA [MHA supplemented with 0.035% hemoglobin (BBL Difco, Sparks, MD, USA, #212392)] and 2% defined growth supplement (IsoVitaleX Enrichment; BBL Difco, Sparks, MD, USA, #211876) for *F. tularensis*. Bacterial cultures with the target turbidity of O.D._630 nm_ = 0.035 for *Y. pestis* and O.D._630 nm_ = 0.1 for *F. tularensis* were used as direct inocula by plating 0.1-ml aliquots on the appropriate agar plate and uniformly spreading the bacterial lawn using a Derigalski spatula. Etest strips of doxycycline and ciprofloxacin (bioMerieux, Marcy-L′Etoile, France) were applied on the plated agar and incubated for 24 h at 28°C for *Y. pestis* or 48 h at 37°C for *F. tularensis*. The MIC was determined by reading the scale values on the strips at the intersection of the growth inhibition zone, according to the manufacturers’ instructions.

## Results

### Growth and Sub-localization of *F. tularensis* in Blood Cultures of Infected Mice

**FIGURE 1:**
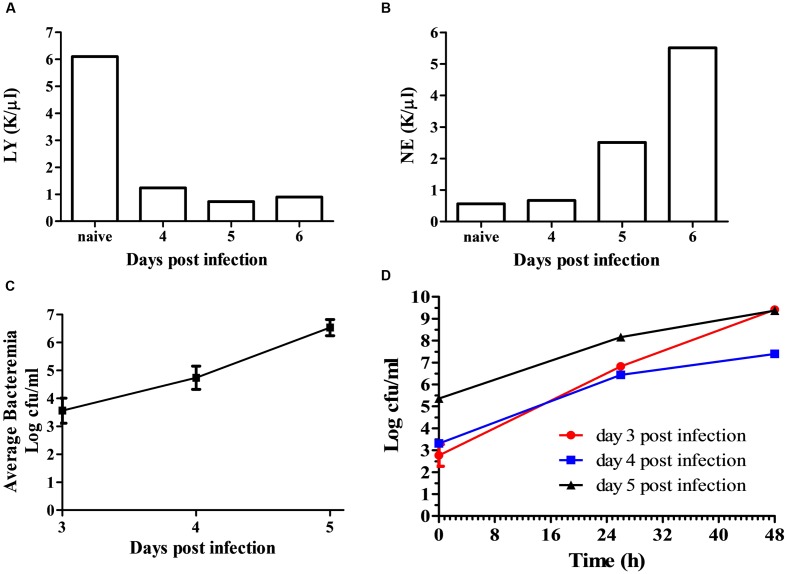
**Bacteremia of *F. tularensis* LVS in the blood of infected mice.** Mice were infected by the intranasal route with 10^5^ CFU per mouse. On days 3–6 post-infection, groups of 5 mice were terminally bled, and the blood was pooled as one sample. Naïve mice served as controls. Blood samples were subjected to clinical analysis, and disease development was indicated by measuring the levels of lymphocytes **(A)** and neutrophils **(B)**. Blood samples were introduced into BACTEC Plus Aerobic/F Culture vial. The levels of bacteria in the blood cultures at time = 0 h were determined by CFU counts and calculated as the *in vivo* bacteremia levels on days 3, 4, and 5 post-infection **(C)**. *F. tularensis* LVS multiplication levels within the blood cultures were determined by monitoring growth at 37°C under moderate agitation (150 rpm) over 48 h. The red line (circle) represents the blood culture at day 3 post-infection, the blue line (square) represents the blood culture at day 4 post-infection, and the black line (triangle) indicates the blood culture at day 5 post-infection **(D)**. All CFU counts were performed in triplicate.

To develop a rapid isolation process, we first characterized the growth of *F. tularensis* in blood cultures and the sub-localization of the bacteria within the blood culture. BALB/c mice were intranasally infected with 10^5^ CFU of *F. tularensis* LVS. Disease development was monitored by daily clinical and physiological examination. Starting on day 3, blood samples were drawn and pooled from 5 mice per day. Neutrophil and lymphocyte counts of samples from days 4 through 6 were compared to pooled blood samples form control naïve mice. The reduction of lymphocyte levels and elevation of neutrophil levels (**Figures 1A,B**) indicated an active disease. The pooled blood was spiked into BACTEC Plus Aerobic/F Culture vials. The CFU counts at time = 0 h in the spiked blood cultures reflected the average *in vivo* bacteremic state. Bacteremia was evident at day 3 post-infection and its level increased with time, demonstrating the involvement of bacteremia in disease progression within the mice in the intranasal infection model. From the volume of the BACTEC Plus Aerobic/F Culture vial content, we calculated that the average concentration of the bacteria in the blood of the infected mice was approximately 5 × 10^3^, 5 × 10^4^, and 5 × 10^6^ CFU/ml on days 3, 4, and 5, respectively (**Figure 1C**). Bacterial growth in the BACTEC Plus Aerobic/F Culture vials were monitored over 48 h. The bacterial load increased by 4–6.5 orders of magnitude over 48 h, regardless of whether blood was drawn at day 3, 4, or 5 (**Figure 1D**). These results suggested that intranasal infection of mice with *F. tularensis* LVS recapitulated the bacteremic phase of the disease and that the bacteria in the blood drawn from the infected animals multiplied in the BACTEC Plus Aerobic/F Culture vials.

*Francisella tularensis* has been reported to reside within blood cells and in the extracellular fraction (Forestal et al., 2007; Yu et al., 2008); thus, it was of interest to examine the distribution of *F. tularensis* LVS within the blood cultures of the infected animals. We employed the SST previously used to separate *Y. pestis* from blood cultures (Steinberger-Levy et al., 2007) and separated the plasma fraction from the cellular fraction. At all time points examined, the majority of *F. tularensis* LVS was observed within the plasma fraction (**Table 1**). Notably, *F. tularensis* LVS was detected in the plasma fraction immediately after blood was withdrawn from the mice, suggesting that the majority of the bacteria *in vivo* were also residing in the plasma and not in the intracellular fraction.

**Table 1:** Localization and growth of *F. tularensis* LVS in blood cultures from infected mice^∗^.

Day of blood withdrawal after infection	Blood culture incubation time (h)	Concentration of bacteria (CFU/ml^∗∗^)	Bacteria in the extracellular fraction (%)
3	0	1.8 × 10^3^ ± 10^2^	85
	26	7.6 × 10^6^ ± 2.5 × 10^6^	100
	48	2.6 × 10^9^ ± 1.5 × 10^8^	68
4	0	2 × 10^4^ ± 1.4 × 10^3^	88
	26	2.7 × 10^6^ ± 2.1 × 10^5^	74
	48	2.5 × 10^7^ ± 2.8 × 10^6^	96
5	0	2.5 × 10^5^ ± 5.5 × 10^4^	N.D.
	24	1.3 × 10^8^ ± 10^7^	92
	48	2.4 × 10^9^ ± 2 × 10^8^	96


*^∗^ Each sample contains pooled blood from five infected mice. ^∗∗^CFU counts were performed in triplicate, and the presented values are the average of the triplicate values. The percent of bacteria in the extracellular fraction was calculated by dividing the CFU counts obtained after separating red blood cells from the plasma fraction using SST by the CFU counts obtained from the blood culture before separation.*

### Growth and Sub-localization of *F. tularensis* in *Ex vivo* Spiked Blood Cultures

To avoid further infection of animals with *F. tularensis* LVS, we performed *ex vivo* spiking human blood cultures with the bacterium. To verify that *F. tularensis* in the *ex vivo* spiked blood cultures also sub-localized in the extracellular fraction, we measured the percentage of plasma-oriented bacteria at various concentrations and time-points post spiking using the SST tubes in a manner identical to that used for bacteria in the blood cultures originating from infected mice (**Table 1**). Moreover, we quantified the percentage of cells associated with *F. tularensis* LVS using both Histopaque gradients and CPT tubes to ensure that the majority of the bacteria indeed resided in the extracellular fraction and not in the cellular fraction (**Table 2**). Similar to the sub-localization in blood cultures from infected mice, the majority of *F. tularensis* LVS was present in the extracellular fraction of the blood in the *ex vivo* spiked blood cultures. These results confirm that *ex vivo* spiking of blood cultures is a valid method for developing procedures to improve the isolation of *F. tularensis* from blood cultures.

**Table 2:** Levels of *F. tularensis* LVS in the mononuclear and the extracellular fractions of spiked naïve blood cultures grown for 1–2 days.

Concentration of bacteria (CFU/ml^∗^)	Bacteria in mononuclear cells (%)		Bacteria in the extracellular fraction (%)
		
	Histopaque gradients	CPT	SST tubes
2.6 × 10^4^ ± 2.6 × 10^3^	10	14	95
2.4 × 10^5^± 4.4 × 10^4^	1.5	2.1	91
9 × 10^7^± 1.4 × 10^7^	16	3	72


*^∗^Samples were plated in triplicate for CFU counts.*

### Comparison of Methods for Rapid Isolation of *F. tularensis* from Blood Cultures

#### Agar Plating and Drop-Plating

**FIGURE 2:**
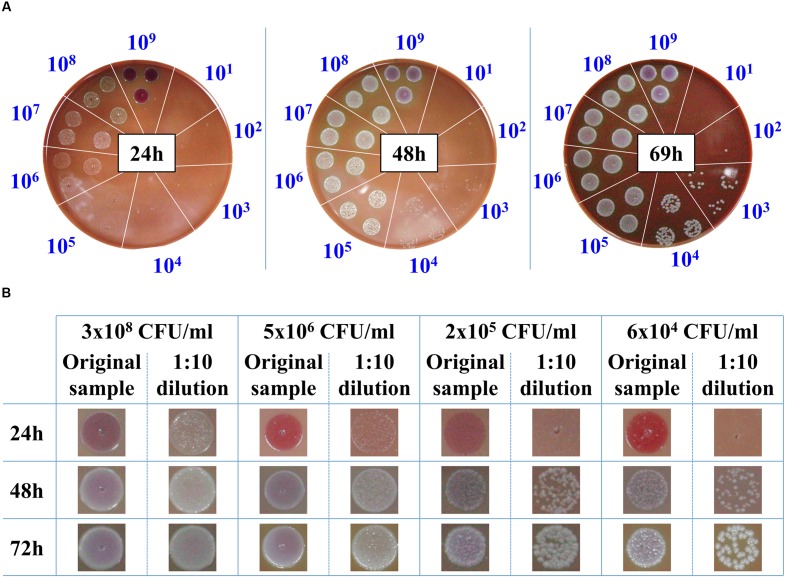
**Drop-plating of *F. tularensis* blood culture samples.** Blood cultures containing *F. tularensis* LVS were grown at 37°C under moderate agitation (150 rpm) to a concentration of approximately 10^9^ CFU/ml. Both direct and tenfold dilutions were drop-plated as 10 μl in triplicate on CHA. The plates were monitored, and growth was recorded at the indicated times **(A)**. Blood cultures of various *F. tularensis* concentrations and their tenfold dilutions were drop-plated. Growth of bacterial lawns and of individual colonies was monitored as a function of time at the indicated incubation time points **(B)**.

The classic procedure to obtain a bacterial culture from a positive blood culture is the isolation of individual colonies on agar plates. These colonies are then used to prepare a standard inoculum for use in AST. Due to the slow growth rates of *F. tularensis* LVS strain, 3 days are required to reach colonies of 1–2 mm to yield sufficient bacteria for AST. Thus, we examined whether drop-plating on CHA to contain the plated bacteria in one spot would shorten the incubation time prior to AST of blood cultures when *F. tularensis* is indicated clinically. For this purpose, blood cultures were spiked with the *F. tularensis* LVS strain and grown to high densities of approximately 10^9^ CFU/ml. The samples were 10-fold diluted, and drops of 10 μl were plated on CHA plates and incubated for the indicated times at 37°C (**Figure 2A**). Following 24 h of incubation, *F. tularensis* LVS drops at -3 log dilution of the original culture (ending with a concentration of approximately 10^6^ CFU/ml) yielded bacterial growth that was sufficient to sample and prepare a standard inoculum for AST assays. Notably, a high drop concentration of 10^9^ CFU/ml plated directly from the blood culture on CHA inhibited the growth of *F. tularensis* LVS, probably due to inhibitory factors within the blood culture. Thus, the diluted sample consistently yielded better growth than the non-diluted sample. The 10-fold dilution of the original sample may have also diluted the inhibitory factors. Sufficient bacterial growth required 48 h for a blood culture spot sample diluted to a concentration of 10^4^ CFU/ml and approximately 3 days when the blood culture was diluted to 10^3^ CFU/ml. To ensure that the growth advantage obtained by drop-plating was not merely due to the dilution of the bacteria, which may also dilute growth inhibitors, we applied the drop-plating procedure with spiked blood cultures of different concentrations (**Figure 2B**). The time schedules obtained for the various blood cultures were within the estimated time frame obtained by the serial dilution of the high-density blood culture (**Figure 2A**). The estimated required incubation times are summarized in **Table 3**. In summary, the drop-plating technique may provide an advantage of 1–2 days of incubation compared to classic plating if the blood culture is pure and contains only *F. tularensis* without other contaminating microorganisms.

**Table 3:** Incubation periods required to obtain 1- to 2-mm colonies of *F. tularensis* LVS following drop-plating as a function of the initial bacterial concentration.

Concentration of bacteria in blood cultures (CFU/ml)^∗^	Estimated time to reach sufficient growth following drop-plating (h)
≥10^8^	∼24
10^7^	∼24
10^6^	∼24-34
10^5^	∼48
10^4^	∼48
<10^4^	∼72


*^∗^Samples were drop-platted in triplicate as described in **Figure 2**.*

#### Combination of SST and Immunomagnetic Separation of *F. tularensis* LVS from Blood Cultures

**FIGURE 3:**
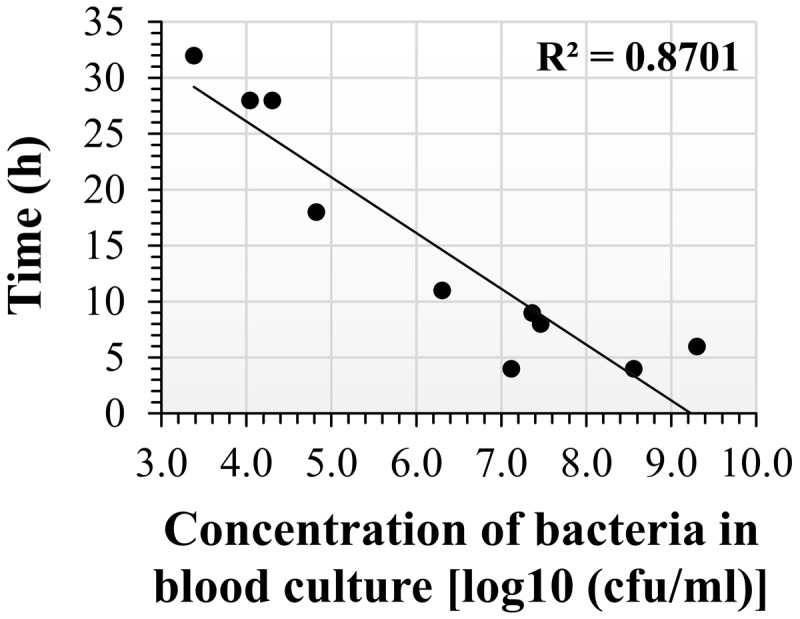
**Incubation time required to reach O.D._630 nm_ = 0.1 following SST-IMS isolation of *F. tularensis* LVS from blood cultures.** *F. tularensis* LVS blood cultures at initial concentrations ranging from 2.4 × 10^3^ CFU/ml to 2.7 × 10^9^ CFU/ml were subjected to the SST-IMS procedure. A 100-μl aliquot of the SST-IMS outputs from each blood culture was plated in 6 replicate in a 96-well microtiter plate. The microtiter plates were incubated in a plate reader for 48 h at 37°C in ambient air, and the absorbance at O.D._630 nm_ was read at 1-h intervals until reaching 0.1 (see dotted line), representing mid-log growth phase. The linear regression of log concentration versus time yielded the following prediction equation: Time (h) = -5 × log (CFU/ml) + 46.

The drop-plating technique reduced the incubation period compared to classic plating, at the expense of a lack of specificity and the potential for a genuine mixed population or fortuitous contamination at blood withdrawal that may remain and be co-cultured in the blood culture. Thus, we used an additional isolation method based on specificity. Of the various available methods, we adopted the SST-IMS procedure. Magnetic beads with a diameter of 50 nm and coated with primary hyperimmune rabbit anti-*F. tularensis* serum were used to selectively capture and enrich *F. tularensis* LVS previously purified from blood cultures by SST. The efficacy of the IMS procedure was at least 60% of the inputs and, in most cases, ranged between 90 and 100%. The IMS outputs were recovered in HLMHI. The growth curves indicated that recovery of the bacteria from the SST-IMS procedure was concentration dependent. We choose O.D._630 nm_ = 0.1 as an endpoint for recovery of *F. tularensis* LVS because this O.D. reflected mid-log phase at all concentrations examined (data not shown) and a bacterial concentration of approximately 2 × 10^9^ CFU/ml. Following the establishment and application of the SST-IMS procedure, we determined the correlation between blood culture concentration and the time to obtain sufficient bacteria for downstream AST procedures. To that end, *F. tularensis* LVS blood cultures ranging from 2.4 × 10^3^ CFU/ml to 2.7 × 10^9^ CFU/ml were subjected to the SST-IMS procedure. The time required to reach the requested 0.1 O.D._630 nm_ is presented in **Figure 3** and **Table 4**. Linear regression of log concentration versus time yielded the prediction equation: Time (h) = -4.9891x log (CFU/ml) + 46.1. At all blood culture concentrations examined, the SST-IMS procedure was superior to drop-plating with respect to time (**Table 3** versus **Table 4**) and specificity.

**Table 4:** Incubation time required to reach O.D._630 nm_ = 0.1 following SST-IMS isolation of *F. tularensis* LVS from blood cultures.

Concentration of bacteria in blood cultures (CFU/ml)	Estimated time to reach sufficient growth following SST-IMS (h)
≥10^8^	4-6
10^7^	5-10
2 × 10^6^	∼12
6.6 × 10^4^	∼18
10^4^	∼28
2.4 × 10^3^	∼33


### Isolation of *Y. pestis* EV76 from Blood Cultures

**FIGURE 4:**
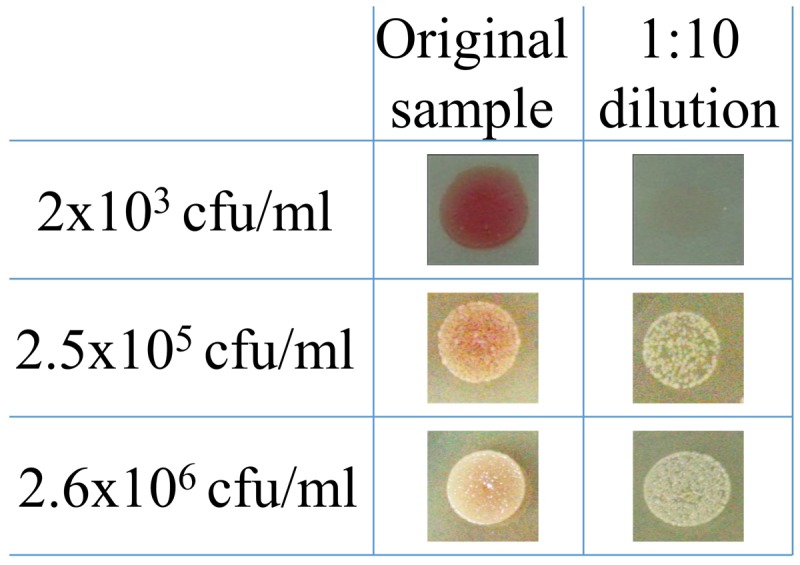
**Drop-plating of *Y. pestis* blood culture samples.** *Y. pestis* EV76 blood cultures were grown at 37°C under moderate agitation (150 rpm) to 2.6 × 10^6^, 2.5 × 10^5^, and 2 × 10^3^ CFU/ml. Both direct and 10-fold dilutions were drop-plated in triplicate as 10-μl aliquots on BHIA. The plates were incubated at 28°C, and growth was recorded 24 h post plating.

**FIGURE 5:**
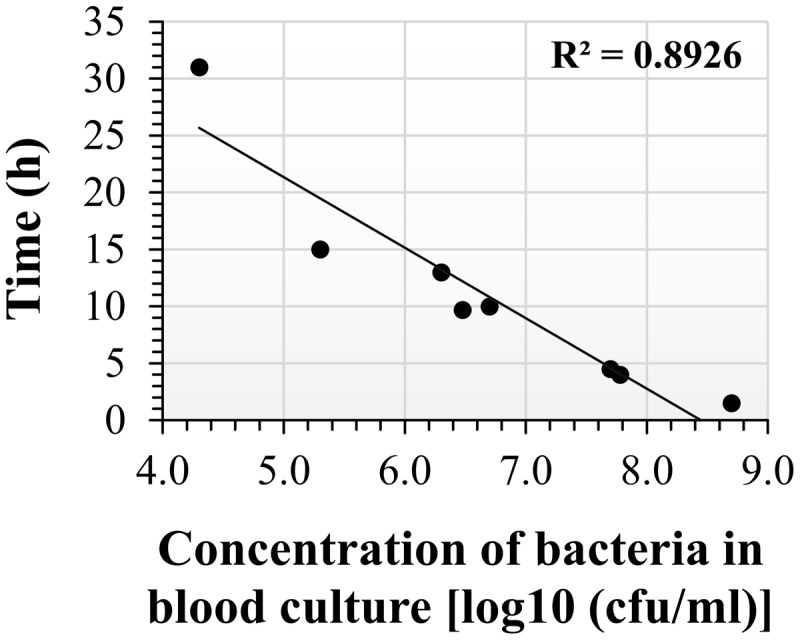
**Incubation time required to reach O.D._630 nm_ = 0.035 following SST-IMS of *Y. pestis* blood cultures.** *Y. pestis* EV76 blood cultures at various initial concentrations were subjected to the SST-IMS procedure. Aliquots of 100 μl of the SST-IMS outputs from each blood culture were plated in 6 replicates in microtiter plates. The microtiter plates were incubated in a plate reader for 48 h at 28°C in ambient air, and the absorbance at O.D._630 nm_ was read at 1-h intervals until reaching 0.035. Linear regression of log concentration versus time yielded the following prediction equation: Time (h) = -6.1926x + 52.311.

We also examined whether SST-IMS is beneficial for timely isolation of bacteria with a faster growth rate. We chose *Y. pestis* because it is also designated as a Tier 1 select agent and the time required for its isolation on agar is approximately 2 days. Thus, a quicker isolation step would be clinically valuable. As with *F. tularensis* LVS, we compared the time periods required for classic plating for colony isolation, drop-plating, and SST-IMS for the isolation of *Y. pestis* EV76 from spiked blood cultures. Two incubation days are required for classic isolation to obtain isolated colonies at any given bacterial concentration. No bacteria were observed on BHIA plates 24 h after drop-plating a blood culture of 2 × 10^3^ CFU/ml (**Figure 4**). Colony formation required 48 h following drop-plating (data not shown). However, at concentrations of 2.5 × 10^5^ CFU/ml and greater, colonies were observed after only 24 h of incubation (**Figure 4**). Thus, the drop-plating technique may provide an advantage of a day compared to classic plating, although at the expense of selectivity. Based on our previous work (Steinberger-Levy et al., 2007), *Y. pestis* EV76 was isolated from the plasma fraction of the blood culture sample using SST and subjected to an IMS procedure using 50-nm magnetic beads coated with primary hyperimmune rabbit anti-*Y. pestis* serum. The efficacy of the IMS procedure was at least 60% of the inputs and ranged between 90 and 100% in most cases. Bacteria were recovered in BHI medium until reaching O.D._630 nm_ = 0.035, reflecting mid-log growth (data not shown) and a bacterial concentration of 2–3 × 10^8^ CFU/ml. The time required to reach O.D._630 nm_ = 0.035 as a function of bacterial concentration is presented in **Figure 5**. Linear regression of log concentration versus time yielded the following prediction equation: Time (h) = -6.1926 × log (CFU/ml) + 52.3. Consistent with the results obtained for *F. tularensis* LVS, SST-IMS was superior to the classic and drop-plating procedures for *Y. pestis* EV76 (**Table 5**). Our results suggest that the SST-IMS procedure is an efficient and prompt procedure that may be used for rapid isolation of the Tier 1 agents *F. tularensis* and *Y. pestis*.

**Table 5:** Comparison of the time schedules for the isolation of *Y. pestis* EV76 from blood cultures by classic plating, drop-plating and SST-IMS.

Concentration of bacteria in blood cultures (CFU/ml)	Incubation time (h)		
	
	Classic plating	Drop-plating^∗^	SST-IMS
10^7^	∼48	∼24	∼8
2.6 × 10^6^	∼48	∼24	∼13
2.5 × 10^5^	∼48	∼24	∼20
2 × 10^4^	∼48	∼24	∼31


*^∗^Samples were drop-plated in triplicate.*

### MIC Determination

The recommended antibiotics for post-exposure prophylaxis of *F. tularensis* and *Y. pestis* are ciprofloxacin and doxycycline (Inglesby et al., 2000; Dennis et al., 2001). As a proof of concept of the validity of our isolation procedure, we performed AST of these recommended antibiotics. *F. tularensis* cultures in HLMHI that reached O.D._630 nm_ = 0.1 (reflecting approximately 2 × 10^9^ CFU/ml) and *Y. pestis* cultures in BHI that reached O.D._630 nm_ = 0.035 (reflecting approximately 2–3 × 10^8^ CFU/ml) were diluted as required by the CLSI to yield approximately 5 × 10^5^–2 × 10^6^ CFU/ml and inoculated into 96-well microtiter plates in a final volume of 100 μl/well. Microdilution tests were performed as described in the Section “Materials and Methods.” The MIC was defined at the end of the incubation time (24 h for *Y. pestis* and 48 h for *F. tularensis*) as the lowest concentration that reduced growth to less than 10% of the O.D._630 nm_ of the growth control, and a lack of visible growth was verified by the unaided eye. MIC values are presented as a range and include all values obtained in repetitions (*n* = 3) (**Table 6**). *F. tularensis* LVS displayed the expected ‘susceptible’ category as indicated by MICs of 0.016 μg/ml for ciprofloxacin, ≤0.5 μg/ml (the endpoint for the susceptible category for this bacterium), and 0.06–0.125 μg/ml for doxycycline, ≤4 μg/ml. Similar MIC ranges were obtained by the Etest method, which does not appear to have an advantage over microdilution with respect to the required incubation period but may prove useful for low levels of contamination in blood cultures. Similarly, microdilution tests (*n* = 3) of *Y. pestis* EV76 revealed MIC values of 0.5–1 μg/ml for doxycycline and 0.016–0.032 μg/ml for ciprofloxacin. In the Etests, the MIC values were similar and ranged from 0.5 to 0.75 μg/ml for doxycycline and 0.023 to 0.032 μg/ml for ciprofloxacin. All MIC results were in complete agreement with assays conducted using standard inoculum and test conditions. These MIC results indicate that the SST-IMS procedure is suitable for the rapid isolation of *F. tularensis* and *Y. pestis* for AST.

**Table 6:** Minimal Inhibitory Concentration (MIC) determination.

	Doxycycline (μg/ml)		Ciprofloxacin (μg/ml)	
		
	Microdilution	Etest	Microdilution	Etest
*F. tularensis* LVS	0.06–0.125	0.125-0.19	0.016	0.008-0.023
*Y. pestis* EV76	0.5–1	0.5–0.75	0.016–0.032	0.023–0.032


## Discussion

The symptoms of tularemia include fever, headache, chills, sore throat, and body aches (Dennis et al., 2001). These symptoms are common to other illnesses, resulting in a low index of suspicion for tularemia among clinicians. Tularemia may be considered an option only after exclusion of more common infectious diseases. Consequently, some tularemia patients may be treated with broad-range antibiotics without actual identification of *F. tularensis* as the causative agent (Fritzsch and Splettstoesser, 2010). Thus, isolation of the bacteria may benefit both identification and, more importantly, AST characterization to assure proper antibiotic treatment. In recent years, efforts have been made to facilitate AST procedures for tularemia and develop new approaches to determine susceptibility toward both the extracellular and intracellular forms of the bacteria (Sutera et al., 2014a; Aloni-Grinstein et al., 2015).

Blood cultures are a potential clinical source of bacteria from the patient. Moreover, determination of bacteremia may have an impact on the clinical outcome and medical consideration toward the patient. For years, bacteremia was rarely identified, but new culture systems have improved growth and detection, and bacteremia is now reported more widely. Indeed, we observed the development of bacteremia in our mouse model during the progression of the disease (**Figure 1**) and determined that the majority of the bacteria were in the plasma fraction rather than the cellular fraction (**Table 1**). In an attempt to mimic blood cultures of infected animals, we *ex vivo* spiked *F. tularensis* LVS into BACTEC Plus Aerobic/F Culture vials containing naïve blood and showed that the bacteria reside mainly in the plasma fraction in spiked blood cultures as well (**Table 2**). This behavior allowed us to develop a rapid isolation procedure for bacteria from blood cultures.

**FIGURE 6:**
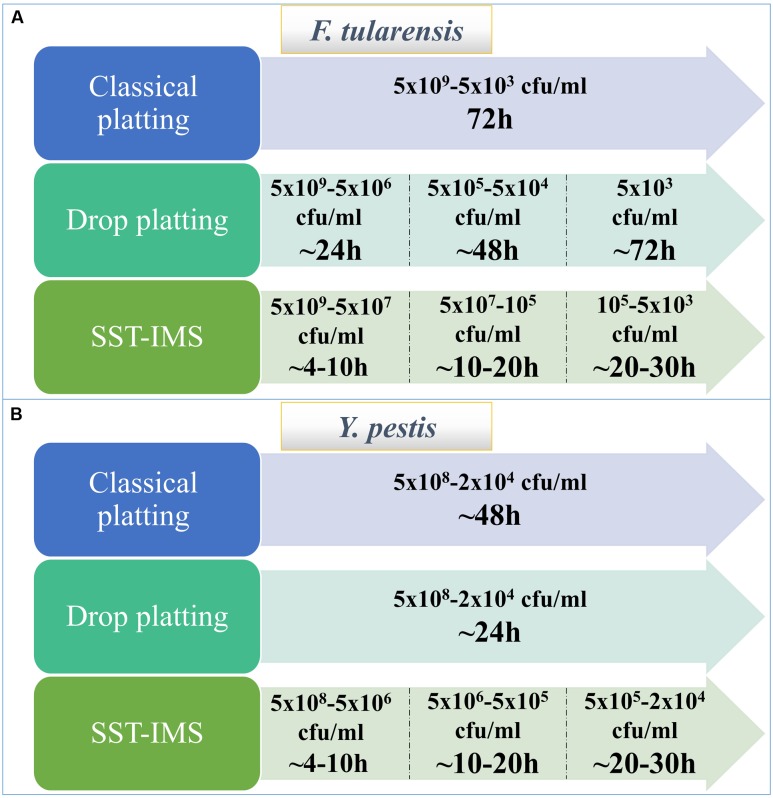
**Time required to reach the required inoculum of bacteria for AST using the different isolation methods.** *F. tularensis* LVS **(A)**. *Y. pestis* EV76 **(B)**.

The isolation of *F. tularensis* from blood cultures is a tedious procedure. Blood culture components negatively influence both PCR identification procedures (Regan et al., 2012) and the prerequisite quantification step and inoculum size adjustment using absorbance determination for standard AST (CLSI, 2015). Thus, sub-culturing is required prior to AST. Due to the slow growth rates of *F. tularensi*s and *Y. pestis*, sub-culturing for isolation is time-consuming. Here, we aimed to bypass the sub-culturing isolation step by either drop-plating or by physical separation from red blood cells and an IMS procedure for transferring the bacteria from the plasma to rich liquid medium. Indeed, drop-plating blood cultures with *F. tularensis* LVS with at least 10^6^ CFU/ml (**Figure 2**) and *Y. pestis* with at least 10^5^ CFU/ml (**Figure 4**) yielded sufficient bacteria by 24 h. However, small percentages of these samples may be contaminated because no selective procedure was applied to distinguish *F. tularensis* or *Y. pestis* from potential contaminating bacteria due to either natural infection or contamination during the blood withdrawal procedure (Weinstein, 2003). This drawback highlights the advantage of IMS, which enriches bacteria by selective immunoseparation. The presence of *F. tularensis* LVS bacteria primarily in the extracellular fraction in blood cultures from infected animals allowed us to obtain the majority of the bacteria from the blood culture by a simple step using SSTs before IMS purification. IMS separates the bacteria from the blood components and growth-inhibitory substances present in the blood culture and components that mask proper quantification by turbidity measurement. The recovered concentrated bacteria in enrichment media permit rapid growth to the essential load necessary for use as an inoculum for various AST methods. Indeed, we have shown that the isolation of bacteria by the SST-IMS procedure permits proper MIC determination by microdilution within a shorter time than the conventional isolation method. Moreover, we have shown that this procedure is superior to classic plating for both *F. tularensis* and *Y. pestis* (**Figure 6**), which represent bacteria with different growth rates. These different bacteria validate the universality of the method for other bacteremia-causing bacteria. In summary, we have shown that the SST-IMS procedure is a rapid, specific procedure for the isolation of bacteria from positive blood cultures. The SST-IMS procedure allows AST for both *F. tularensis* and *Y. pestis* to be performed within a significantly shorter time period after the blood culture is identified as positive for these pathogens (**Tables 3**–**5**), thus shortening the time necessary to conduct AST for proper antibiotic treatment determination.

## Author Contributions

Research project design: RA-G, OS, SY, IS-L, and RB. Experiments: RA-G, OS, SY, MA, SM, and RB. Writing: RA-G and RB.

## Conflict of Interest Statement

The authors declare that the research was conducted in the absence of any commercial or financial relationships that could be construed as a potential conflict of interest.

## Footnotes

1 https://www.selectagents.gov/selectagentsandtoxinslist.html
